# The Prognostic Role of Spot Urinary Sodium and Chloride in a Cohort of Hospitalized Advanced Heart Failure Patients: A Pilot Study

**DOI:** 10.3390/life13030698

**Published:** 2023-03-05

**Authors:** Andrew Xanthopoulos, Charalambos Christofidis, Chris Pantsios, Dimitrios Magouliotis, Angeliki Bourazana, Ioannis Leventis, Niki Skopeliti, Evangelia Skoularigki, Alexandros Briasoulis, Grigorios Giamouzis, Filippos Triposkiadis, John Skoularigis

**Affiliations:** 1 Department of Cardiology, University Hospital of Larissa, 41100 Larissa, Greece; 2 Michigan Society of Thoracic and Cardiovascular Surgery Quality Collaborative, Ann Arbor, MI 48105, USA; 3 Department of Therapeutics, Faculty of Medicine, National and Kapodistrian University of Athens, 11527 Athens, Greece

**Keywords:** urinary, spot, sodium, chloride, advanced heart failure, loop diuretics, prognosis

## Abstract

Recent studies have demonstrated the prognostic value of spot urinary sodium (UNa^+^) in acutely decompensated chronic HF (ADCHF) patients. However, data on the prognostic role of UNa^+^ and spot urinary chloride (UCl^−^) in patients with advanced HF are limited. In the present prospective pilot study, we examined the predictive value of UNa^+^ and UCl^−^ concentration at baseline, at 2 h and at 24 h after admission for all-cause mortality and HF rehospitalization up to 3 months post-discharge. Consecutive advanced HF patients (n = 30) admitted with ADCHF and aged > 18 years were included in the study. Loop diuretics were administered based on the natriuresis-guided algorithm recommended by the recent HF guidelines. Exclusion criteria were cardiogenic shock, acute coronary syndrome, estimated glomerular filtration rate < 15 mL/min/1.73 m^2^, severe hepatic dysfunction (Child–Pugh category C), and sepsis. UNa^+^ at baseline (Area Under the Curve (AUC) = 0.75, 95% Confidence Interval (CI) (0.58–0.93), *p* = 0.019) and at 2 h after admission (AUC = 0.80, 95% CI: 0.64–0.96, *p* = 0.005) showed good and excellent discrimination, respectively. UCl^−^ at 2 h after admission (AUC = 0.75, 95%CI (0.57–0.93), *p* = 0.017) demonstrated good discrimination. In the multivariate logistic regression analysis, UNa^+^ at 2 h (*p* = 0.02) and dose of loop diuretics at admission (*p* = 0.03) were the only factors independently associated with the study outcome. In conclusion, UNa^+^ and UCl^−^ may have a prognostic role in hospitalized advanced HF patients.

## 1. Introduction

Acute heart failure (AHF) refers to the rapid or gradual onset of symptoms and/or signs of HF, severe enough for the patient to seek urgent medical intervention, leading to an unplanned hospital admission or an emergency department presentation [1]. Congestion, a typical finding in acute decompensated chronic HF (ADCHF), refers to signs and symptoms of extracellular fluid accumulation that result in increased cardiac filling pressures [2]. Since sodium (Na^+^) and water retention in the extracellular space are responsible for the increase in venous return and cardiac filling pressures, intravenous loop diuretics are used to ameliorate symptoms of fluid overload in patients with ADCHF [3,4]. In particular, loop diuretics inhibit the Na^+^-K^+^-2Cl^−^ symporter at the ascending loop of Henle and have the most potent diuretic effect, promoting the excretion of Na^+^ and chloride (Cl^−^) [5,6]. Therefore, not surprisingly, loop diuretics form the backbone of diuretic therapy in ADCHF, being used in over 90% of patients [7].

Traditionally, the estimation of decongestion is based on the findings from the clinical examination (symptoms and/or signs), urine output, weight loss, blood levels of natriuretic peptides and renal function (i.e., creatinine/urea), which is not optimal [8,9]. According to the latest guidelines, the dose of intravenous loop diuretics should be adjusted based on the spot urinary Na^+^ (UNa^+^) concentration (natriuresis-guided treatment) in order to be achieved timely and successful decongestion [1]. It has been reported that early treatment with intravenous loop diuretics is associated with lower in-hospital mortality in AHF [10]. In this regard, a number of recent studies have demonstrated the prognostic value of UNa^+^ concentration in patients presenting in the emergency department (ED) with ADCHF [9,11,12,13,14], while less is known about the spot urinary chloride (UCl^−^) [15,16]. Nevertheless, data on the role of UNa^+^ and UCl^−^ concentration in advanced HF patients, a population of HF patients who exhibit frequent rehospitalizations and poor survival, are limited [17,18]. Furthermore, the appropriate use of diuretics in advanced HF remains challenging since those patients frequently have low blood pressure, deteriorating renal function, diuretic resistance and electrolyte disturbances.

Starting from the idea that the early risk stratification of advanced HF patients may result in better classification of those patients, timely administration of decongestive therapies and improved outcomes, the present pilot study investigated the prognostic value of spot urinary electrolytes (Na^+^ and Cl^−^), at various time points (i.e., at admission before the administration of loop diuretics, at 2 and 24 h after the administration of diuretics) and their association with unfavorable clinical events in a small cohort of advanced HF patients hospitalized for ADCHF.

## 2. Materials and Methods

### 2.1. Study Population

**Figure 1:**
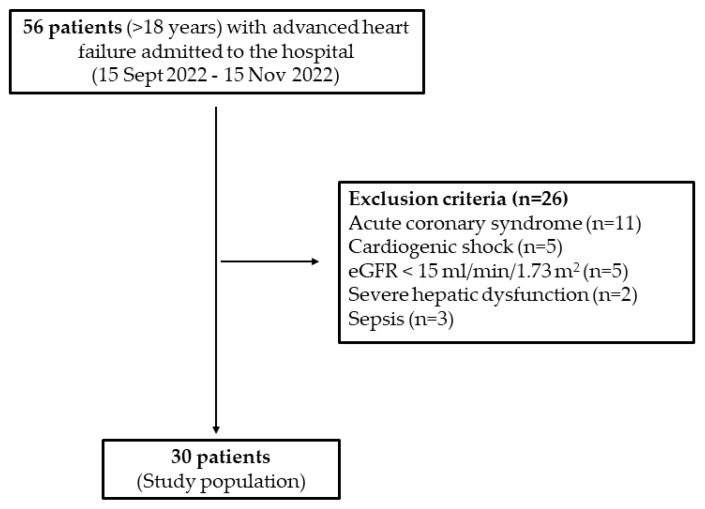
Study flowchart.

Consecutive patients hospitalized for ADCHF in a tertiary University Hospital from 15 September 2022 to 15 November 2022 and aged > 18 years were included in the study. Exclusion criteria were cardiogenic shock, acute coronary syndrome, estimated glomerular filtration rate < 15 mL/min/1.73 m^2^, severe hepatic dysfunction (Child–Pugh category C), and sepsis (Figure 1). All patients enrolled were on a natriuresis-guided algorithm recommended by the recent HF guidelines [1]. UNa^+^ and UCl^−^ were collected at baseline (before the administration of loop diuretics) with the use of urine catheter, at 2 h after the loop diuretic administration (2 h after admission) and thereafter at various time points during hospitalization based on the abovementioned algorithm.

The evaluation of the patients at admission included clinical assessment, laboratory blood and urine tests, as well as echocardiography. UNa^+^ and UCl^−^ were measured with the use of the Roche Hitachi cobas 8000 (cobas ISE) on samples obtained at different time points of hospitalization. N-terminal pro-b-type natriuretic peptide (NT-proBNP) was measured with the use of Radiometer’s AQT90 FLEX immunoassay analyzer, while blood gas with GEM PREMIER 3000 Analyzer (Instrumentation Laboratory). Blood tests were measured with the use of the Roche Hitachi cobas 8000 (cobas c 702) on samples obtained for standard-of-care evaluation. Finally, echocardiography was performed within 1 h after admission in accordance with current recommendations, with the use of eSaote MyLabX6 echo machine [19]. The left ventricular ejection fraction (LVEF) was calculated with the use of two-dimensional echocardiography by implementing the biplane method of disks summation technique [19]. The loop diuretic used in the present study was furosemide.

This study conformed to the principles outlined in the Declaration of Helsinki and was approved by the Ethics Committee of the University of Thessaly (protocol code: 349). All patients provided written informed consent.

### 2.2. Definitions

Advanced HF was defined based on the following criteria despite optimal medical treatment [1]:

1. Severe and persistent symptoms of HF [NYHA class III (advanced) or IV] within the last 6 months;
2. LVEF ≤ 30%;
3. Persistently high (or increasing) BNP or NT-proBNP values and severe left ventricular diastolic dysfunction or structural abnormalities;
4. Episodes of pulmonary or systemic congestion requiring high-dose i.v. diuretics (or diuretic combinations) or episodes of low output requiring inotropes or vasoactive drugs or malignant arrhythmias causing >1 unplanned visit or hospitalization in the last 12 months.

If chronic HF deteriorates, either suddenly or slowly, the episode may be described as ‘decompensated’ HF. This can result in hospital admission [1]. HF hospitalization was defined as a hospitalization requiring at least an overnight stay in hospital caused by onset or substantive worsening of HF symptoms and/or signs requiring the augmentation (an increase in the dose or frequency of administration) of oral medications or new administration of intravenous (IV) HF therapy, including inotropes, diuretics or vasodilators [20]

### 2.3. Outcomes

The study outcome combined all-cause mortality and/or HF rehospitalization. The study follow-up was 3 months post-discharge.

### 2.4. Statistical Analysis

The normality of the data was assessed using D’Agostino–Pearson test. A two-tailed unpaired *t*-test and Mann–Whitney U-test were performed for parametric and nonparametric continuous data, respectively. A chi-square test was performed for categorical variables. We assessed the discrimination (i.e., the ability to separate those who did from those who did not die/rehospitalized) of the urine electrolytes (Na^+^ and Cl^−^) at baseline, after 2 and 24 h. Discrimination was assessed by generating receiver-operating characteristic (ROC) curves and by calculating the area under the ROC curve (AUC). The AUC was determined by calculating the 95% confidence intervals and compared using nonparametric paired tests, as described by DeLong et al. [21]. We defined poor, good and excellent model discrimination with the AUC of <0.70, 0.70–0.79 and 0.80–1.00, respectively [21]. Repeated measures analysis of variance (ANOVA) was conducted in order to explore the changes in Na and CL over the follow-up period. Bonferroni correction was used for the pairwise time comparisons. A logistic regression analysis was performed for the events. In order to find factors independently associated with prognosis, multiple logistic regression was conducted in a stepwise manner with all-cause mortality or HF rehospitalization at 3 months as dependent variable. Differences were considered significant (rejection of the null hypothesis) with a *p* < 0.05. All data were analyzed using Microsoft^®^ Excel 365 16.66.1 (Microsoft, Redmond, Washington, DC, USA) and Prism^®^ Graphpad 9.5.0 for Mac (GraphPad Software, San Diego, CA, USA) as well as SPSS 28 (IBM Corp. Released 2021. IBM SPSS Statistics for Windows, Version 28.0. Armonk, NY, USA: IBM Corp)

## 3. Results

### 3.1. Baseline Characteristics

**Figure 2:**
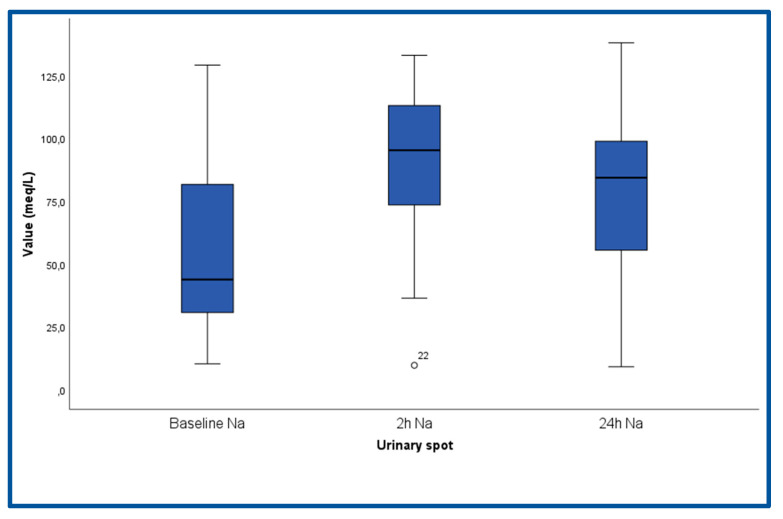
Box plots of spot urinary Na^+^ at baseline (admission), 2 h and 24 h after admission.

**Figure 3:**
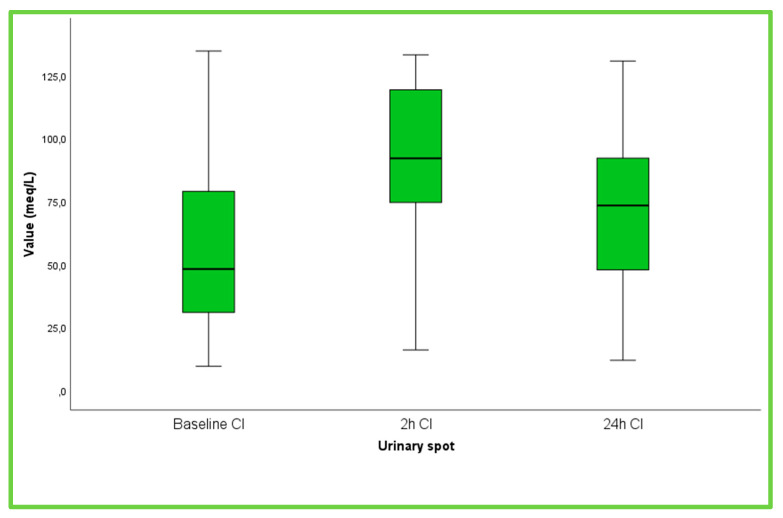
Box plots of spot urinary Cl^−^ at baseline (admission), 2 h and 24 h after admission.

The characteristics of the study population are presented in Table 1. The cohort consisted of elderly patients (mean age 73 years), whereas half of them (n = 15, 50%) were females. The majority (70%) of patients were in New York Heart Association (NYHA) III, while the rest were in NYHA IV, and the mean NT-proBNP was approximately 13,320 pg/mL, mirroring the advanced stages of HF. Renal function was mildly to moderately impaired, whereas hematocrit, hemoglobin and blood electrolytes were within the normal range. The mean baseline left ventricular ejection fraction was low (37.3%), and the inferior vena cava was dilated (24 mm). Regarding medical treatment, the majority of patients were on β-blockers and loop diuretics, whereas approximately half of them were on ACE inhibitors/ARBs, ARNis or MRAs. The box plots of UNa^+^ and UCl^−^ and the urine output at various time points are depicted in Figure 2, Figure 3 and Figure S1, respectively. UNa^+^ and UCl^−^ had significant changes over the follow-up period (P_ANOVA_ < 0.001). More specifically, after Bonferroni correction, it was found that at 2 h, both UNa^+^ and UCl^−^ were higher compared to their values at admission (*p* < 0.001 and *p* < 0.001, respectively) and at 24 h (*p* = 0.020 and *p* = 0.050, respectively).

### 3.2. Study Outcomes

The combined study outcome (all-cause death and HF rehospitalization) was met in 15 (50%) patients during the study follow-up.

**Figure 4:**
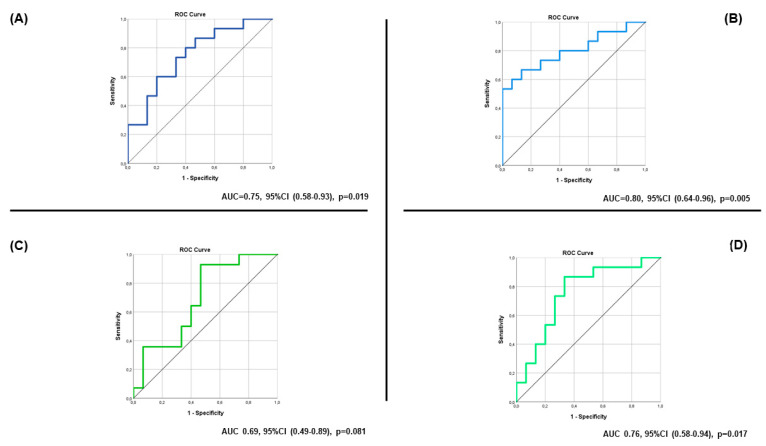
Receiver operating curve (ROC) for the combined endpoint of all-cause mortality and HF rehospitalization (**A**) for the admission spot urinary Na^+^, (**B**) for spot urinary Na^+^ at 2 h, (**C**) for the admission spot urinary Cl^−^, (**D**) for spot urinary Cl^−^ at 2 h.

UNa^+^ at baseline (area under the curve (AUC) = 0.75, 95% Confidence Interval (CI) (0.58–0.93), *p* = 0.019) and at 2 h after loop diuretic administration (AUC = 0.80, 95% CI (0.64–0.96), *p* = 0.005) showed good and excellent discrimination, respectively (Figure 4). On the contrary, UNa^+^ at 24 h was not of prognostic value (AUC 0.74, 95%CI (0.50–0.97), *p* = 0.056) (Figure S2).

Interestingly, the optimal cut-off value for the admission UNa^+^ was ≤49 meq/L, with 73.3% sensitivity and 66.7% specificity. Patients with UNa^+^ ≤ 49 meq/L exhibited 5.5 times higher risk for the study outcome compared to those with UNa^+^ > 49 meq/L (Odds Ratio = 5.50, 95% CI (1.15–26.41), *p* = 0.033). The optimal cut-off value for the UNa^+^ at 2 h was ≤95.5 meq/L, with 73.3% sensitivity and 73.3% specificity. Patients with UNa^+^ at 2 h ≤95.5 had 7.56 times higher risk of all-cause mortality or HF rehospitalization compared to those with UNa^+^ at 2 h >95.5 meq/L (Odds Ratio = 7.56, 95% CI (1.50–38.15), *p* = 0.014).

UCl^−^ at 2 h after admission (AUC = 0.76, 95%CI (0.58–0.94), *p* = 0.017) demonstrated good discrimination (Figure 4). The optimal cut-off value for the UCl^−^ at 2 h was ≤99.8 meq/L, with 86.7% sensitivity and 66.7% specificity. Patients with UCl^−^ at 2 h ≤ 99.8 meq/L had 13 times higher odds for all-cause mortality or HF rehospitalization compared to those with UCl^−^ at 2 h >99.8 meq/L (Odds Ratio = 13, 95% CI (2.08–81.48), *p* = 0.006). On the contrary, UCl^−^ at baseline (AUC 0.69, 95% CI (0.49–0.89), *p* = 0.081) and at 24 h (AUC 0.66, 95% CI (0.43–0.90), *p* = 0.193) was not of prognostic significance (Figure 4 and Figure S3). The homoscedasticity plots are shown in Figures S4 and S5.

A univariate logistic regression analysis revealed the factors that were associated with the study outcome (Table 2). These were the UNa^+^ at admission (*p* = 0.02) and at 2 h (*p* = 0.01), the UCl^−^ at 2 h (*p* = 0.02), urine output at 6 h (*p* = 0.01) and the dose of loop diuretic at admission (*p* = 0.01). The multivariate logistic regression analysis, in a stepwise manner, revealed that UNa^+^ at 2 h and loop diuretic dose at admission were the only independent factors for the study outcome (Table 3).

## 4. Discussion

The current study is the first to provide concurrent insight into the predictive value of UNa^+^ and UCl^−^ as a response to diuretic treatment in advanced decompensated hospitalized HF patients who were on the natriuresis-guided therapy algorithm. Main findings may include the following (1) UNa^+^ at baseline showed good discrimination, (2) UNa^+^ 2 h after admission demonstrated excellent discrimination and was independently associated with the study outcome, (3) UCl^−^ at 2 h after admission demonstrated good discrimination for the combined outcome of all-cause mortality and rehospitalization during the follow up (3 months) for hospitalized patients with advanced HF, (4) UNa^+^ and UCl^−^ at 2 h after the administration of iv loop diuretics were both significantly higher compared to their values at admission.

There is increasing evidence that higher urinary Na^+^ concentration during treatment for acute HF is related to a better prognosis, higher possibility of achieving euvolemia and shorter hospitalizations [11,22]. However, few data are available for Na^+^ excretion during an acute decompensation of patients with advanced HF. The present study attempted to highlight the significance of electrolyte status and associated neurohormonal activation in the fragile advanced HF patient. In particular, it showed that the administration of high-dose loop diuretics at admission (mean dose of furosemide 118.0 mg) led to significant UNa^+^ and UCl^−^ increase at 2 h, compared to their baseline values. Diuretic resistance (a failure to increase fluid and Na+ output sufficiently to relieve volume overload despite escalating doses of a loop diuretic) is a frequent finding among advanced HF patients and it is associated with poor prognosis [23,24]. Therefore, timely identification (with the use of spot urine electrolytes) of patients with the highest risk of HF rehospitalization and death may lead to earlier and more intense decongestive therapy and better outcomes.

There is evidence that the assessment of UNa^+^ concentration in acute HF is a more accurate prognostic marker than urine output. Interestingly, in the present study, urine output collected at 6 and 24 h after admission was not associated independently with the study outcomes. So far, Testani et al. demonstrated that UNa^+^ at 1–2 h corresponds to the total Na^+^ concentration in a 6-h urine collection, allowing for a prompt assessment of response to diuretic treatment and individualized titration [25]. Similarly, Collins et al. underscored the predicting utility of early evaluation of natriuresis (total urine Na^+^), 1 h after diuretic administration, in correspondence to the clinical outcome of in-hospital worsening HF [12]. Our protocol included assessment of baseline urine Na^+^ (at admission) as well as evaluation at the predetermined point of 2 h after the loading dose of diuretics. It is one of few studies to evaluate baseline levels of urinary Na^+^ as well as early Na^+^ excretion, both demonstrating efficacy as discriminating factors. The good prognostic ability of low levels of baseline urinary Na^+^ comes in agreement with a previous study by Martens et al. that underscored low levels of urinary Na^+^ in chronic HF patients as a means of foreseeing acute decompensation [26].

To our concern, so far, only one prospective study concerning UNa^+^ and response to diuretic treatment in advanced HF was conducted, however, including only ambulatory HF patients in a short surveillance time. Spot urine samples were obtained at first voided urine after loading dose of diuretics and compared to total urine output at three hours, and the values were correlated to 30-day hospitalization or emergency department visit. Specifically, a cut-off of 65 mmol/h and a urine output of less than 1200 mL were associated with 69% rate of hospitalization in 30 days [17]. Our study included patients with decompensated advanced HF, and urinary Na^+^ was evaluated at admission and at 2 h and 24 h after administration of loop diuretics. Insertion of the urinary catheter at all patients at admission in our protocol and subsequent evaluation of UNa^+^ at 2 h excluded the possibility of pre-diuretic residual urine, yet differentiating it from the aforementioned study.

It could be anticipated that linear UNa^+^ excretion in advanced HF patients, who typically come up with longer hospitalizations, would correlate to clinical outcome, duration of efficient decongestion and mortality. Nevertheless, UNa^+^ at 24 h failed to demonstrate a further discrimination value (AUC = 0.74, *p* = 0.056), reinforcing the position that timely and tailored administration of loop diuretics is of utmost importance regarding all-cause mortality and rehospitalizations for advanced HF patients. This observation fills a knowledge gap, as data on advanced HF natriuresis is scarce due to a lack of studies, and emphasizes the value of prompt optimal dosing. Interestingly, UNa^+^ at 2 h and initial diuretic dose administration were the only independent factors for the study outcome.

The evaluation of UNa^+^ as a biomarker of response to HF treatment has been gaining ground in the last few years [4]. In the present study, we attempted to evaluate the excretion of electrolytes in a critically ill patient with advanced HF. Taking into consideration that conventional markers such as glomerular filtration rate (GFR) or NT-proBNP have failed to correspond to acute HF outcome, we purposely attempted to incorporate a biomarker that correlates to water and extracellular volume handling without being affected by glomerular function, which may be variously aggravated in advanced HF patients. In the present study, both markers of renal function (i.e., urea and creatinine), as well as NT-proBNP, were not associated with the study outcome.

While longitudinal profiles of UNa^+^ in hospitalized HF patients have been evaluated, little is currently known concerning the prognostic significance of UCl^−^ [14,27]. Cl^−^ is among the key electrolytes that participate in fluid homeostasis. Although so far neglected, Cl^−^ is the main regulator of the macula densa, the region of renal juxtaglomerular apparatus that senses NaCl and fluid status [28,29]. It should be emphasized that the action of Cl^−^ on the lately detected with-no-lysine (K)- WNK protein kinase evolves in enhanced water and electrolytes reabsorption via upregulation of the Na^+^-K^+^-Cl^−^ cotransporter, possibly elucidating a mechanism of diuretic resistance in HF [30]. Our study shows for the first time that UCl^−^ derived soon after loop diuretic administration can discriminate patients with advanced HF at high risk for ominous clinical outcomes. Specifically, a cut-off value of ≤99.8 meq/L distinguished patients with 13 times higher odds of all-cause mortality or HF rehospitalization in 3 months. Our findings are in agreement with a recent small study highlighting serum and urine Cl^−^ indices as an even better estimator of neurohormonal activation than Na^+^ indices in acute HF, correlating firmly to plasma renin activity [31].

Sodium–glucose co-transporter-2 inhibitors (SGLT-2) inhibitors, a novel drug class, inhibit the SGLT-2 receptors predominantly expressed in the proximal tubule of the nephron; thus, they induce glycosuria and natriuresis and have been shown to reduce the combined endpoint of all-cause mortality and HF rehospitalization in chronic HF patients irrespective of the LVEF [32,33,34,35,36]. However, recent studies revealed that these drugs might also be beneficial in hospitalized HF patients. In particular, the EMPA-RESPONSE-AHF study showed that the SGLT-2 inhibitor empagliflozin was safe, increased urinary output and reduced a combined endpoint of worsening HF, rehospitalization for HF or death at 60 days, compared to placebo [37]. Furthermore, the randomized EMPULSE demonstrated that more patients treated with empagliflozin had a clinical benefit compared with placebo (stratified win ratio, 1.36; *p*  =  0.0054), meeting the primary endpoint (a hierarchical composite of all-cause death, number of HF events and time to first HF event, or a 5 point or greater difference in change from baseline in the Kansas City Cardiomyopathy Questionnaire Total Symptom Score at 90 days) [38]. Nevertheless, an interesting recent meta-analysis of two observational and six randomized studies reported conflicting results concerning the true efficacy of SGLT-2 inhibitors in acute HF patients, including “hard” surrogate endpoints [32]. Whether the UNa^+^ can be used as a prognostic marker and its potential cut-off values in patients already receiving SGLT-2 inhibitors needs to be investigated in future studies. Interestingly, in the present pilot study, 16.7% of patients were on SGLT-2 inhibitors at admission.

All patients enrolled in the present study were on a natriuresis-guided therapy algorithm. In other words, the administration of loop diuretics, as well as their dosage, was based on the UNa^+^ values [1,5]. Interestingly, the pragmatic urinary sodium-based treatment algoritHm in acute heart failure (PUSH-AHF) trial will reveal whether natriuresis-guided therapy, using a pre-specified stepwise diuretic treatment approach, improves natriuresis and clinical outcomes in patients with acute HF [39]. From the perspective of the clinician, a two-dimensional, contemporaneous assessment with two biomarkers (UNa^+^ and UCl^−^) that both demonstrate a good discriminatory ability can minimize the possibility of a random finding while being timesaving and easily obtainable.

Patients with advanced HF have different characteristics from those of the ADCHF population [40,41,42]. For example, an analysis from the Acute Decompensated Heart Failure National Registry (ADHERE) revealed that patients with advanced HF tended to be younger (69.6 vs. 72.8 years), were more often males (65% vs. 49%) and were more likely to have hyperlipidemia/dyslipidemia (65% vs. 41%) and coronary artery disease (73% vs. 57%) compared to those with ADCHF [40]. Furthermore, in patients with advanced HF, symptoms appear to be more related to fatigue and less to fluid status/volume overload than in patients with ADCHF [40]. Lastly, a patient with advanced HF may often exhibit episodes of ADCHF, but not all patients with ADCHF have advanced HF.

## 5. Study Limitations

This study has some limitations: (a) The present work was a non-randomized, single-center study, and therefore, the risk of bias and confounding cannot be excluded, despite multiple adjustments. However, the current study was prospective in nature, and the main advantage of prospective over retrospective cohort and case-control studies is that baseline exposure status is correctly assessed, not only recalled, reducing the risk of selection bias [43]. Furthermore, all patients were on the same natriuresis-guided therapy. (b) The number of patients enrolled was relatively small; however, the present was a pilot study, patients were closely monitored, and none was lost during follow-up. (c) Currently, there is no universal definition of advanced HF [44]. In the present work, advanced HF was defined based on recent guidelines [1]. Lastly, only 16.7% of patients were on SGLT-2 inhibitors at admission, mirroring the currently low global prescription rate of this drug category in HF populations [45,46,47].

## 6. Conclusions

Recent studies have reported the prognostic value of UNa^+^ in ADCHF patients, while less is known about the role of UCl^−^ in the same population of patients. The present pilot work adds to the existing literature by demonstrating that UNa^+^ and UCl^−^ may predict the combined short-term outcome of all-cause mortality and HF rehospitalization in a small cohort of hospitalized advanced HF patients who followed the same loop diuretic treatment algorithm. UNa^+^ at 2 h after admission was associated independently with prognosis. Whether early risk stratification of advanced HF patients with the use of UNa^+^ and UCl^−^ leads to better outcomes needs to be elucidated in the future. Larger studies are urgently needed.

## Figures and Tables

**Table 1:** Characteristics of the study population.

Demographics	Number of Patients, n = 30
Female, n (%)	15 (50)
Mean age, years (SD)	73 (13)
Sodium Consumption, n (%)	28 (100)
Systolic arterial pressure, mmHg (SD)	126 (25)
Diastolic arterial pressure, mmHg (SD)	77 (17)
Heart rate	88 (21)
NYHA class, n (%)	
I	0 (0)
II	0 (0)
III	21 (70)
IV	9 (30)
Comorbidities	
Hypertension, n (%)	9 (30)
Diabetes Mellitus, n (%)	8 (26.7)
Coronary Artery Disease, n (%)	8 (26.7)
Dyslipidemia, n (%)	5 (16.7)
Valvular disease (at least moderate), n (%)	6 (20)
Arterial blood gases	
pH	7.44 (0.06)
pO_2_, mm Hg (SD)	63 (20)
pCO_2_, mm Hg (SD)	42 (10)
HCO_3_, mmol/L (SD)	20 (8)
Lactate, mmol/L (SD)	1.3 (1.1)
Blood tests	
Hematocrit, % (SD)	38.5 (6.8)
Hemoglobin, g/dL (SD)	12.2 (2.3)
Platelets, K/μL (SD)	261 (100)
Prothrombin Time, sec (SD)	19.4 (9.3)
International Normalized Ratio (SD)	1.6 (0.8)
Activated Partial Thromboplastin Clotting Time, s (SD)	31 (5)
Fibrinogen, mg/dL (SD)	344 (81)
C-reactive protein, mg/dL (SD)	2 (3)
Glucose, mg/dL (SD)	137 (47)
Urea, mg/dL (SD)	80 (50)
Creatinine, mg/dL (SD)	1.4 (0.7)
K^+^, mmol/L (SD)	4.6 (0.5)
Na^+^, mmol/L (SD)	135.4 (6.9)
NT-proBNP, ng/L (SD)	13319.6 (9577.2)
Iron, mg/dL (SD)	106.6 (63.7)
Ferritin, ng/mL (SD)	159.2 (134.4)
Urine collection	
Baseline (admission)	
Spot Na^+^, meq/L (SD)	55.9 (34.5)
Spot Cre, mg/dL (SD)	18.5 (15.3)
Spot Cl^−^, meq/L (SD)	90.7 (28.4)
At 2 h	
Spot Na^+^, meq/L (SD)	90.2 (30.4)
Spot Cre, mg/dL (SD)	19.4 (15.5)
Spot Cl^−^, meq/L (SD)	90.6 (28.3)
At 24 h	
Spot Na^+,^ meq/L (SD)	78.98 (30.58)
Spot Cre, mg/dL (SD)	44.73 (36.24)
Spot Cl^−^, meq/L (SD)	71.35 (32.37)
Urine output at 6 h, mL (SD)	1676.67 (616.9)
Urine output at 24 h, mL (SD)	3985.42 (1384.5)
Cardiac echo	
LVEDD, mm (SD)	54.8 (8.9)
LVEF %, (SD)	37.3 (15)
Left Atrium, mm (SD)	51.4 (6.6)
TDI RV S _wave velocity,_ cm/sec (SD)	9.2 (1.7)
RVSP, mm Hg (SD)	51 (14)
IVC, mm (SD)	24 (4.5)
Medical treatment	
b-blocker, n (%)	24 (80)
ACE-inhibitor/ARB/ARNI, n (%)	16 (53.3)
MRA, n (%)	12 (40)
Loop Diuretic, n (%)	23 (76.7)
Dose of loop diuretic (furosemide) at admission (mg)	118.0 (80.7)
SGLT-2 inhibitor, n (%)	5 (16.7)

NYHA: New York Heart Association, N-terminal pro-b-type natriuretic peptide: NT-proBNP, LVEDD: left ventricular end–diastolic diameter, LVEF: left ventricular ejection fraction, TDI: tissue doppler imaging, RV: right ventricle, RVSP: right ventricular systolic pressure, IVC: inferior vena cava, ACE: angiotensin-converting enzyme, ARB: angiotensin receptor blockers, ARNi: angiotensin receptor-neprilysin inhibitors, MRA: mineralocorticoid receptor antagonists, SGLT-2 inhibitor: Sodium–glucose Cotransporter-2 inhibitor.

**Table 2:** Univariate logistic regression.

Variable	Odds Ratio	95% CI	*p*-Value
Age	1.04	0.98, 1.10	0.21
Systolic arterial pressure	0.99	0.97, 1.03	0.95
Diastolic arterial pressure	0.97	0.92, 1.02	0.24
NYHA III	0.37	0.07, 1.92	0.23
Heart rate	0.96	0.91, 1.00	0.05
Hypertension	1.37	0.28, 6.60	0.69
Diabetes Mellitus	4.33	0.70, 26.53	0.11
Coronary Artery Disease	1.00	0.19, 5.04	1.00
Dyslipidemia	5.09	0.49, 52.28	0.17
Valvular disease (at least moderate)	1.00	0.16, 5.98	1.00
pH	0.39	0.30, 0.55	0.89
pO_2_	1.00	0.96, 1.04	0.98
pCO_2_	1.06	0.97, 1.19	0.24
HCO_3_	1.09	0.96, 1.32	0.31
Lactate	1.20	0.53, 3.76	0.68
Hct	1.04	0.94, 1.17	0.46
Hgb	1.09	0.78, 1.54	0.61
Plt	1.00	0.99, 1.00	0.61
PT	1.07	0.97, 1.22	0.23
INR	2.22	0.71, 11.15	0.23
APTT	1.07	0.91, 1.27	0.41
FIB	0.99	0.98, 1.00	0.22
CRP	0.88	0.63, 1.15	0.39
Glucose	1.00	0.99, 1.02	0.76
Urea	1.01	1.00, 1.04	0.11
Creatinine	1.55	0.53, 5.31	0.44
K	0.76	0.15, 3.52	0.73
Na	0.92	0.80, 1.02	0.16
NT-proBNP	1.00	1.00, 1.00	0.27
Iron	1.00	0.994, 1.01	0.35
Ferritin	0.99	0.99, 1.00	0.33
**Baseline Spot Na**	0.97	0.94, 0.99	**0.02**
**Spot Na 2 h**	0.95	0.91, 0.99	**0.01**
Spot Na 24 h	0.98	0.95, 1.01	0.20
Baseline Spot Cre	1.04	0.99, 1.12	0.20
Spot Cre 2 h	1.05	0.98, 1.13	0.10
Spot Cre 24 h	0.99	0.96, 1.01	0.55
Baseline Spot Cl	0.97	0.95, 1.0	0.06
**Spot Cl 2 h**	0.96	0.92, 0.99	**0.02**
Spot Cl 24 h	0.98	0.96, 1.01	0.33
**Urine output 6 h**	0.99	0.996, 0.999	**0.01**
Urine output 24 h	1.00	0.99, 1.00	0.16
LVEDD	1.02	0.91, 1.16	0.71
LVEF	1.00	0.95, 1.05	0.90
Left Atrium	1.08	0.93, 1.30	0.37
TDI RV	0.46	0.18, 0.88	0.05
RVSP	1.01	0.96, 1.07	0.65
IVC	1.04	0.88, 1.24	0.67
b-blocker	2.36	0.36, 15.45	0.36
ACE-inhibitor/ARB/ARNI	1.00	0.23, 4.19	1.00
MRA	3.14	0.68, 14.50	0.14
Loop Diuretic	-	-	0.99
**Dose of loop diuretic at admission**	1.01	1.00, 1.02	**0.01**
SGLT-2 inhibitor	5.09	0.49, 52.28	0.17

NYHA: New York Heart Association, N-terminal pro-b-type natriuretic peptide: NT-proBNP, LVEDD: left ventricular end–diastolic diameter, LVEF: left ventricular ejection fraction, TDI: tissue doppler imaging, RV: right ventricle, RVSP: right ventricular systolic pressure, IVC: inferior vena cava, ACE: angiotensin-converting enzyme, ARB: angiotensin receptor blockers, ARNi: angiotensin receptor–neprilysin inhibitors, MRA: mineralocorticoid receptor antagonists, SGLT-2 inhibitor: Sodium–glucose Cotransporter-2 inhibitor.

**Table 3:** Multivariate logistic regression.

Variable	B	OR	95% CI	*p*-Value
Spot Na 2h	−0.04	0.95	0.91, 0.99	**0.02**
Loop diuretic dose at admission	0.01	1.01	1.00, 1,02	**0.03**

## Data Availability

Data are available by the authors upon reasonable request.

## Supplementary Materials

The following supporting information can be downloaded at: https://www.mdpi.com/article/10.3390/life13030698/s1, Figure S1: Box plots of the urine output at 6 and 24 h after admission; Figure S2: Spot urinary Na^+^ at 24 h. (AUC 0.74, 95%CI (0.50–0.97), *p* = 0.056); Figure S3: Spot urinary Cl^−^ at 24 h. (AUC 0.66 (0.42–0.89), *p* = 0.193); Figure S4: Homoscedasticity plot for spot urinary Na+ at various time points. E: events, NE: non-events; Figure S5: Homoscedasticity plot for spot urinary Cl^−^ at various time points. E: events, NE: non-events.
