# Supplementary material for: The Prognostic Role of Spot Urinary Sodium and Chloride in a Cohort of Hospitalized Advanced Heart Failure Patients: A Pilot Study
Source: Life (Basel). 2023 Mar 5;13(3):698. doi: 10.3390/life13030698 (PMC10054455; doi:10.3390/life13030698)
Supplement: Supplementary file 1 [file life-13-00698-s001.zip › life-2213077-supplementary.pdf]

Supplementary Material

The prognostic role of spot urinary sodium and chloride in a cohort of hospitalized advanced heart failure patients: A pilot study

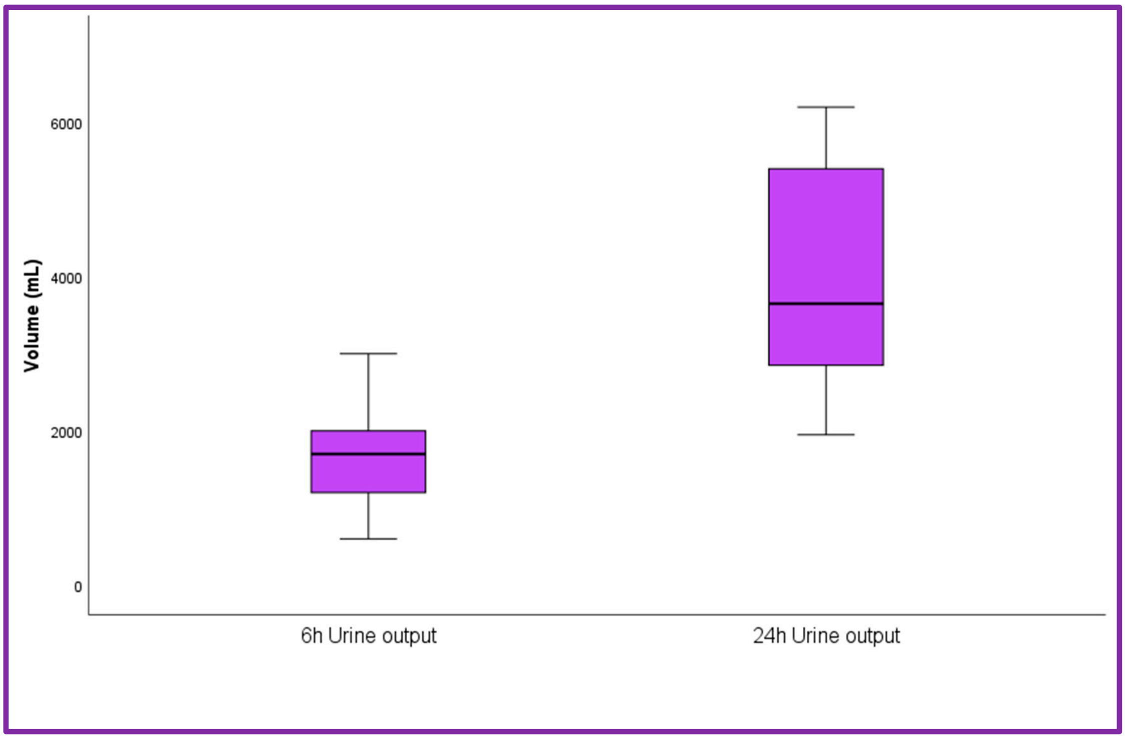

Figure S1. Box plots of the urine output at 6 and 24 hours after admission

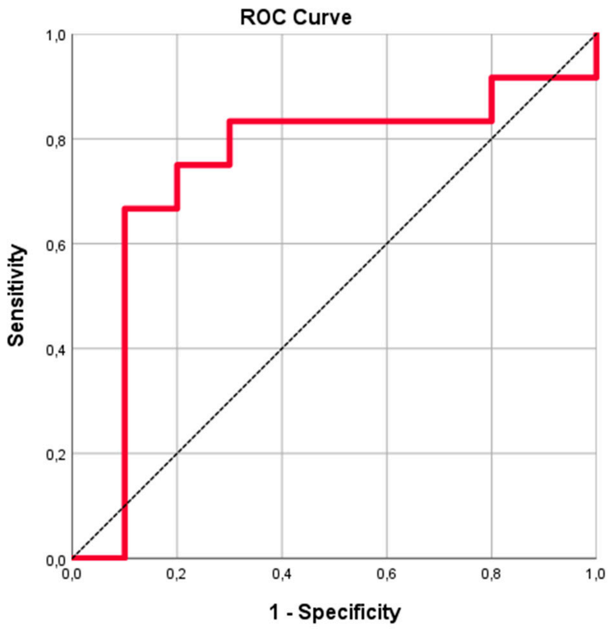

Figure S2. Spot urinary Na^+ at 24 hours. **[**AUC 0.74, 95%CI (0.50-0.97), p=0.056]

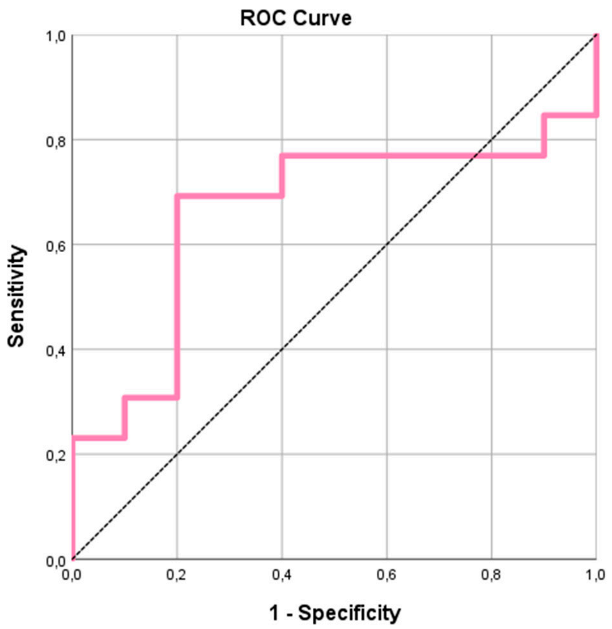

Figure S3. Spot urinary Cl^- at 24 hours. [AUC 0.66 (0.42-0.89), p=0.193]

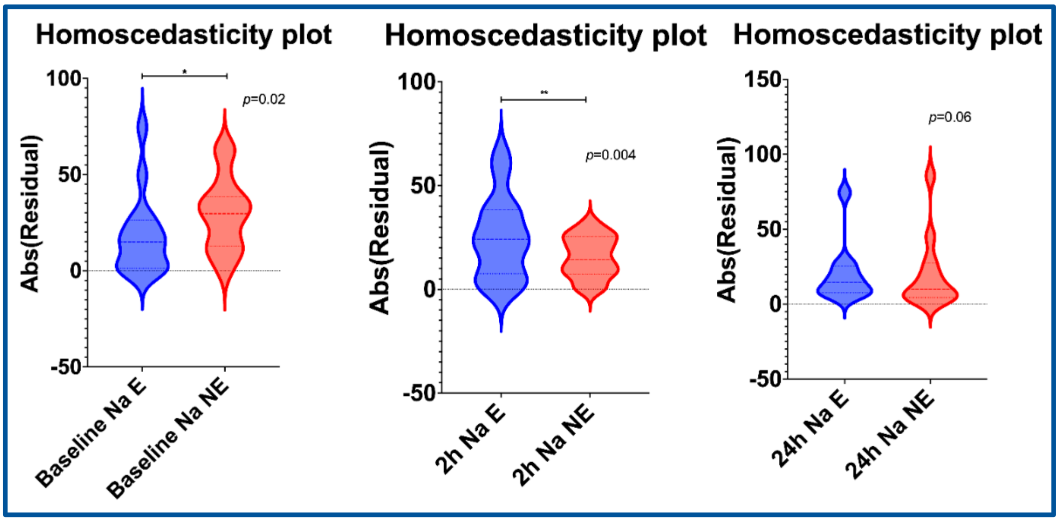

Figure S4. Homoscedasticity plot for spot urinary Na+ at various time points.
E: events, NE: non-events

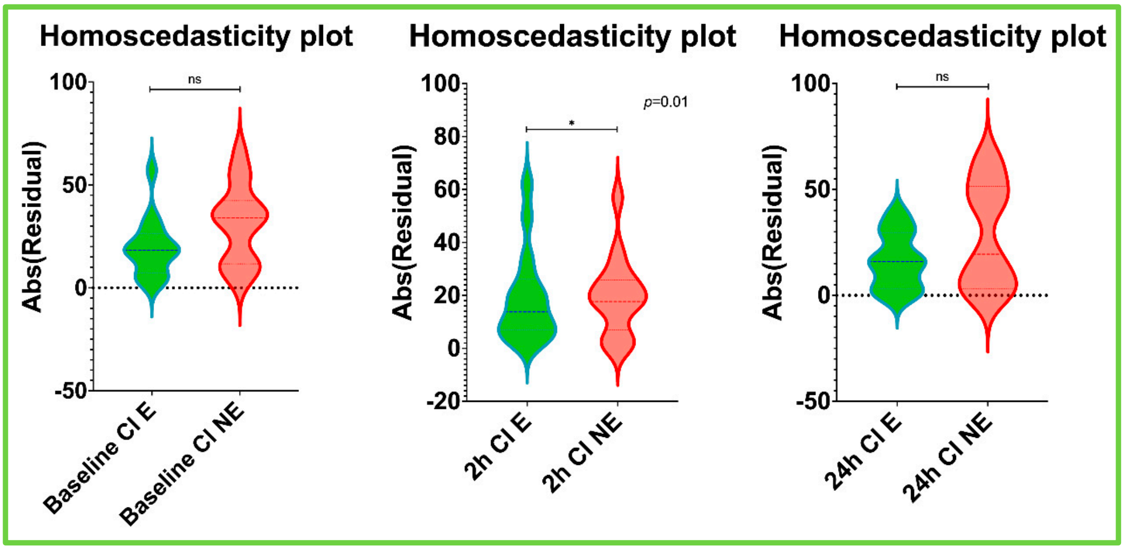

Figure S5. Homoscedasticity plot for spot urinary Cl- at various time points.
E: events, NE: non-events
